# CD99 Modulates the Proteomic Landscape of Ewing Sarcoma Cells and Related Extracellular Vesicles

**DOI:** 10.3390/ijms25031588

**Published:** 2024-01-27

**Authors:** Alessandra De Feo, Marcello Manfredi, Caterina Mancarella, Joaquín J. Maqueda, Veronica De Giorgis, Ymera Pignochino, Marika Sciandra, Camilla Cristalli, Massimo Donadelli, Katia Scotlandi

**Affiliations:** 1 Laboratory of Experimental Oncology, IRCCS Istituto Ortopedico Rizzoli, 40136 Bologna, Italy; alessandra.defeo@ior.it (A.D.F.); caterina.mancarella@ior.it (C.M.); joaquin.juradomaqueda@ior.it (J.J.M.); marika.sciandra@ior.it (M.S.); camilla.cristalli@ior.it (C.C.); 2 Department of Translational Medicine, University of Piemonte Orientale, 28100 Novara, Italy; marcello.manfredi@uniupo.it (M.M.); veronica.degiorgis@uniupo.it (V.D.G.); 3 Department of Clinical and Biological Sciences, University of Turin, 10043 Turin, Italy; ymera.pignochino@ircc.it; 4 Sarcoma Unit, Candiolo Cancer Institute, FPO-IRCCS, 10060 Turin, Italy; 5 Department of Neurosciences, Biomedicine and Movement Sciences, Section of Biochemistry, University of Verona, 37134 Verona, Italy

**Keywords:** extracellular vesicles, Ewing sarcoma, heterogeneity, proteomic, mass spectrometry, biomarkers

## Abstract

Ewing sarcoma (EWS) is an aggressive pediatric bone tumor characterized by unmet clinical needs and an incompletely understood epigenetic heterogeneity. Here, we considered CD99, a major surface molecule hallmark of EWS malignancy. Fluctuations in CD99 expression strongly impair cell dissemination, differentiation, and death. CD99 is also loaded within extracellular vesicles (EVs), and the delivery of CD99-positive or CD99-negative EVs dynamically exerts oncogenic or oncosuppressive functions to recipient cells, respectively. We undertook mass spectrometry and functional annotation analysis to investigate the consequences of CD99 silencing on the proteomic landscape of EWS cells and related EVs. Our data demonstrate that (i) the decrease in CD99 leads to major changes in the proteomic profile of EWS cells and EVs; (ii) intracellular and extracellular compartments display two distinct signatures of differentially expressed proteins; (iii) proteomic changes converge to the modulation of cell migration and immune-modulation biological processes; and (iv) CD99-silenced cells and related EVs are characterized by a migration-suppressive, pro-immunostimulatory proteomic profile. Overall, our data provide a novel source of CD99-associated protein biomarkers to be considered for further validation as mediators of EWS malignancy and as EWS disease liquid biopsy markers.

## 1. Introduction

Ewing sarcoma (EWS) is an aggressive bone and soft-tissue tumor of mesenchymal origin, mostly affecting children and young adults, with a peak incidence in the second decade of life. The tumor presents a high tendency to metastasize, with most patients harboring micrometastases and around 30% of patients showing detectable metastasis at diagnosis, predominantly in the lungs and/or bone/bone marrow. For these patients, the survival rate is still dismal. Indeed, while the current standard treatment regimen, which includes systemic adjuvant and neo-adjuvant chemotherapy, surgery and/or radiotherapy, has increased the survival rate to 70% in patients with localized disease, the survival rate of patients presenting metastases at diagnosis remains as low as 30% [1].

From the genetic point of view, EWS is well characterized. In all cases, EWS is driven by chimeric transcription factors encoded by FET::ETS fusion oncogenes, most commonly Ewing sarcoma breakpoint region 1 protein (EWSR1)::Friend leukaemia integration 1 transcription factor (FLI1) (EWS::FLI1; 85% of cases) [2]. EWS::FLI1 has both transcription-activating and -repressive functions, which are critical for successful oncogenesis (for a review, see [3]), and is responsible for the epigenetic rewiring of EWS’s genetic landscape. Besides transcription, recent evidence supports its role as a critical determinant of epigenetic plasticity and tumor cell phenotype [4,5,6], since it regulates the chromatin architecture [7], RNA splicing [8], R-loop formation [9], and protein translation [2,10].

Although several early studies have clearly shown that EWS::FLI1 is the oncogenic driver of this tumor, its expression is considered a necessary but not sufficient condition to fully sustain EWS aggressiveness. In particular, *STAG2* mutations, which are found in 15–21% of tumors [11,12], impact on EWS::FLI1 activity and induce a remodulation of oncogenic and developmental transcriptional programs to enhance migration and metastasis [7,13]. In addition, EWS cells are also characterized by the peculiarly high expression of CD99, a cell membrane protein involved in many essential cellular functions, including cell death and differentiation, migration and cell adhesion, intracellular protein trafficking, endocytosis, and exocytosis [14,15]. While EWS::FLI1 sustains CD99 expression [16,17], CD99 is required to maintain EWS aggressiveness independent of the chimera. EWS cells expressing EWS::FLI1 but silenced for CD99 show a reduction in cell growth, migration, and metastasis formation and an increase in neural differentiation [17,18]. Fluctuations in the CD99 levels in EWS cells are associated with specific transcripts [19] and microRNA [20] expression profiles, but also with actin remodeling [21], the spatial redistribution of critical regulators of cancer aggressiveness, such as zyxin [19], Rock2 [21], and ERK1/2 [22], and the modulation of intracellular signaling pathways including Src signaling [23]. In addition, CD99 is released by EWS cells through extracellular vesicles (EVs), where it serves a role in the selective sorting of non-coding RNAs [18,20]. EVs represent lipid vesicles released by cells into the extracellular space [24]. EVs transfer their cargo, including mRNAs, miRNAs, and proteins, to recipient cells, thus influencing their biological responses [24,25]. We previously demonstrated that EVs derived from EWS cells deprived of CD99 reflect the experience of the cell of origin, becoming relevant messengers and powerful propagators of cellular status [18,20]. Thus, beyond its role in the intracellular compartment, CD99 also has a role in cellular communication, contributing to the heterogeneity of EWS.

In this study, we applied proteomics technology and bioinformatic analysis to systematically characterize the consequences of CD99 modulation in the EWS proteome. We considered both the intracellular and the extracellular compartments to explore common and distinctive CD99-associated proteomic signatures in EWS cells and related EVs. Proteomics can serve as a valuable tool to elucidate novel mechanisms underlying the phenotypic heterogeneity of EWS and to gain novel insight into the identification of biomarkers associated with EWS aggressiveness.

## 2. Results

### 2.1. Proteomics Analysis and Functional Annotations of CD99-Associated Proteins in EWS Cells

**Figure 1:**
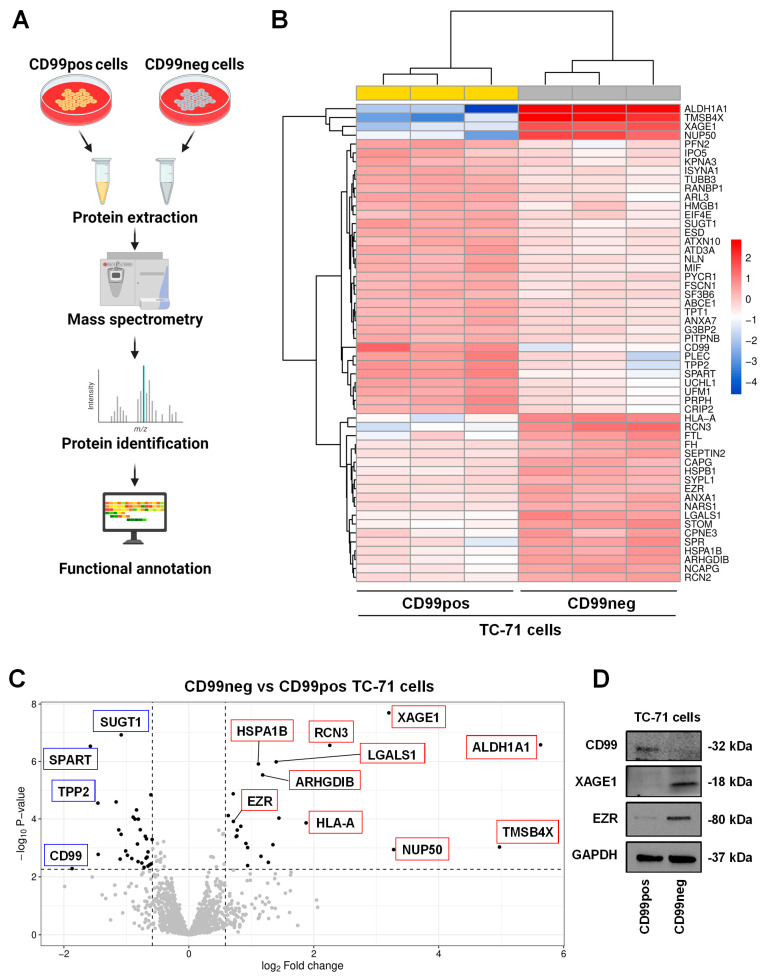
CD99 modulates the proteomic landscape of EWS cells. (**A**) Scheme of the experimental plan for proteomic analysis performed in CD99neg versus CD99pos EWS cells. The figure was created with BioRender.com (accessed on 15 November 2023). (**B**) Heatmap of differentially expressed proteins in CD99neg versus CD99pos TC-71 cells. (**C**) Volcano plot showing the identified differentially expressed proteins in CD99neg compared to CD99pos TC-71 cells. The *x* axis represents the fold change (FC) (log_2_) in protein expression in CD99neg cells versus CD99pos cells, and the *y* axis represents the −log_10_ *p*-value. Dotted lines indicate fold-change cutoffs (|log_2_(1.5)| and *p*-value cutoff, corresponding to a FDR < 0.05 −log_10_(0.0053)). Black points on the left indicate down-regulated proteins; black points on the right indicate up-regulated proteins; and gray points indicate proteins without significant differential expression. Biologically relevant top up-regulated (red boxes) and down-regulated (blue boxes) proteins are marked within the graph. (**D**) Representative Western blot depicting the expression of CD99, XAGE1, and EZR in TC-71 CD99pos and CD99neg cells. GAPDH was used as a loading control.

We took advantage of a stable CD99-depleted experimental TC-71 model previously generated via the short hairpin (shRNA) approach [17,18,19,20] and we performed mass spectrometry (MS) on control-transfected (CD99pos) and CD99-depleted (CD99neg) TC-71 cells (Figure 1A). A total of 1343 and 1356 proteins were found in CD99pos and CD99neg cells, respectively. Based on the differential expression analysis of proteins between CD99pos and CD99neg cells, we identified a signature of 54 proteins (23 up-regulated and 31 down-regulated in CD99neg vs. CD99pos cells; Table 1).

The differential expression profile is depicted using a hierarchical clustering heatmap (Figure 1B), which shows that CD99neg cells are characterized by a distinct proteomics signature compared to CD99pos cells. Moreover, the volcano plot, a type of scatterplot that shows statistical significance (*p*-value) versus magnitude of change (fold change), enables a quick visual identification of the most biologically significant proteins associated with CD99 (Figure 1C). Among the most up-regulated proteins in CD99neg cells, we found aldehyde dehydrogenase 1A1 (ALDH1A1), thymosin beta-4 (TMSB4X), nuclear pore complex protein Nup50 (NUP50), X antigen family member 1 (XAGE1), reticulocalbin-3 (RCN3), and the HLA class I histocompatibility antigen, A alpha chain (HLA-A) (Table 1). On the contrary, among the most down-regulated proteins in CD99neg cells, we found CD99, confirming the accuracy of our experimental model, spartin (SPART), tripeptidyl-peptidase 2 (TPP2), plectin (PLEC), ubiquitin carboxyl-terminal hydrolase isozyme L1 (UCHL1), peripherin (PRPH), and profilin-2 (PFN2) (Table 1). The mass spectrometry results were validated for selected up- (XAGE1, EZR) and down-regulated (CD99) proteins via Western blotting (Figure 1D).

**Figure 2:**
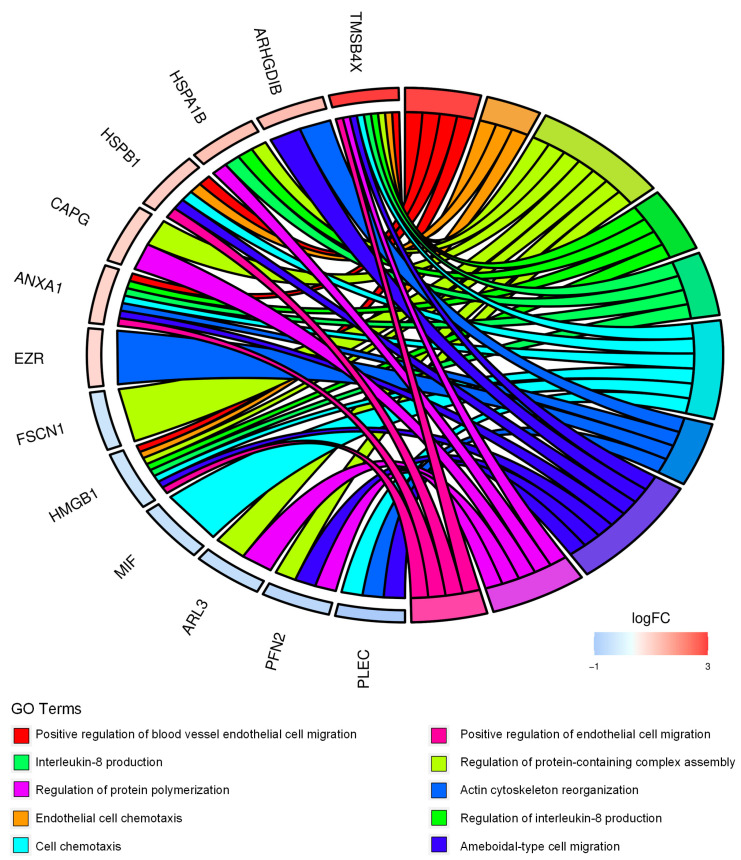
CD99-associated protein signature points to cell migration and immune-modulation in EWS cells. Chord diagram displays the relationship between the 54 differentially expressed proteins in CD99neg versus CD99pos TC-71 cells and the most significant GO biological processes. LogFC represents the fold change (FC) (log_2_) in protein expression in CD99neg cells versus CD99pos cells.

We then performed functional annotation of the 54 differentially expressed proteins using Bioconductor packages. The analysis identified cell chemotaxis, actin cytoskeleton reorganization, ameboidal-type cell migration, and interleukin-8 production among the most significant biological processes (Figure 2, Table S1).

Overall, the results indicate that CD99neg cells display proteomic changes associated with cytoskeleton remodeling and immunostimulatory/inflammatory processes.

### 2.2. Identification and Functional Annotation of Shared Proteins between CD99pos/neg Cells and Related EVs

**Figure 3:**
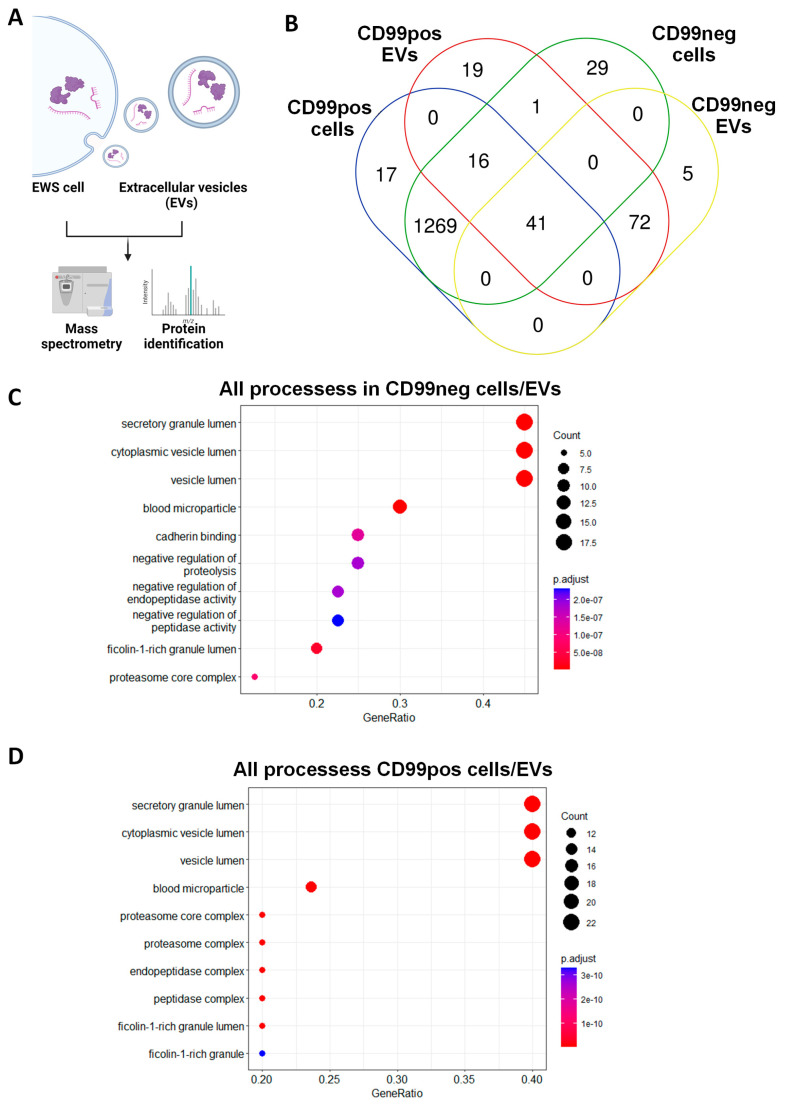
CD99pos/neg cells share a variety of proteins with related EVs. (**A**) Scheme of the experimental plan for mass spectrometry analysis performed in EWS cells and related EVs. The figure was created with BioRender.com (accessed on 15 November 2023). (**B**) Venn diagram showing the overlap of proteins between CD99pos and CD99neg cells and related EVs. (**C**) Functional annotation of the 41 cell/EV common proteins in the CD99neg variant and (**D**) the 57 cell/EV common proteins in the CD99pos variant.

We performed MS analysis of CD99pos and CD99neg cell-derived EVs and we compared them to the related cellular proteomic profile (Figure 3A). Nanosight confirmed the EVs’ size while Western blotting analyses confirmed the presence of EV markers including ALIX and TSG101 (Supplementary Figure S1). In CD99pos EVs, we identified a total 149 proteins. Of those, 57 (38.2%) were shared with related EWS cells, while 92 (61.7%) proteins were exclusively enriched in EVs (Figure 3B). In CD99neg EVs, we identified a total of 118 proteins. Of those, 41 (34.7%) were shared with related EWS cells, while 77 (65.2%) proteins were exclusively enriched in the EVs (Figure 3B). We performed functional annotation of the 41 cell/EV common proteins in CD99neg and of the 57 cell/EV common proteins in the CD99pos variant using Bioconductor software version 3.14. In the CD99neg and CD99pos models, the analysis identified secretory/cytoplasmic/vesicle lumen processes, neutrophil degranulation, and post-translational protein modification (Figure 3C,D; Tables S2 and S3). Interestingly, we observed an enrichment in proteasome-associated processes in the CD99pos variant (Figure 3D).

### 2.3. Identification and Functional Annotation of CD99-Associated Proteins in EVs

**Figure 4:**
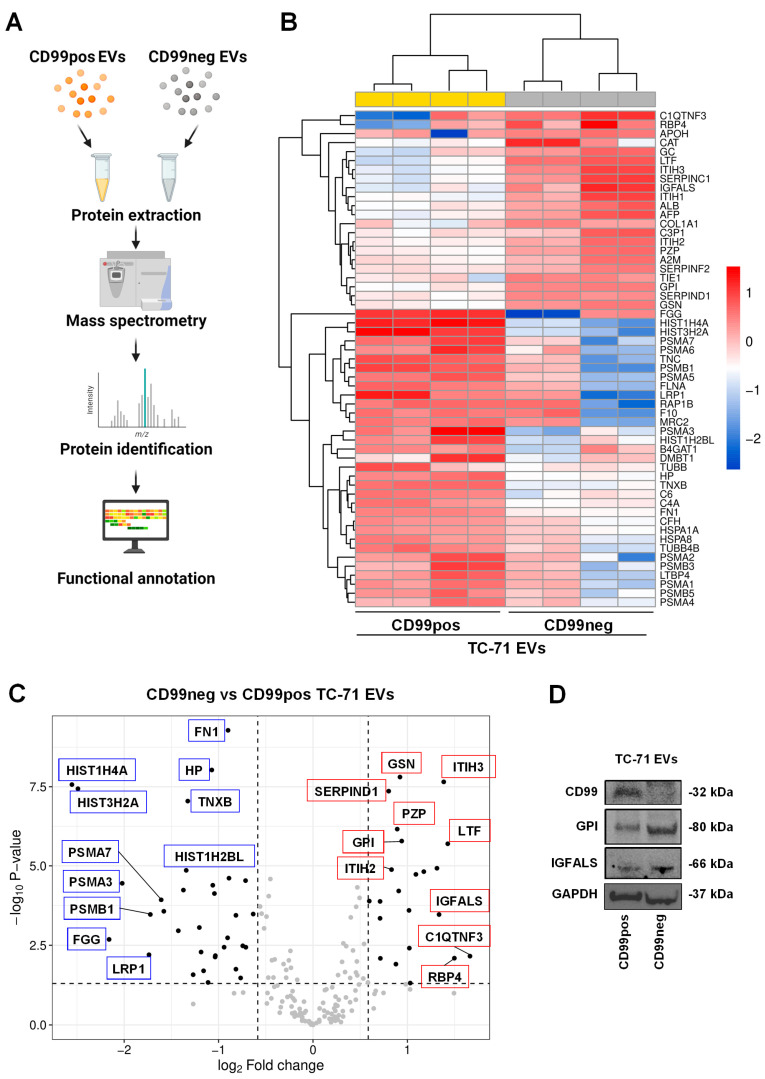
CD99 modulates the proteomic landscape of EWS EVs. (**A**) Schematic of the experimental plan for proteomic analysis performed in EVs extracted from CD99pos versus CD99neg EWS cells. The figure was created with BioRender.com (accessed on 15 November 2023). (**B**) Heatmap of differentially expressed proteins in CD99neg versus CD99pos EVs extracted from the TC-71 experimental model. (**C**) Volcano plot showing the identified differentially expressed proteins in CD99neg compared to CD99pos EVs. The *x* axis represents the fold change (FC) (log_2_) of protein expression in CD99neg cells versus CD99pos cells, and the *y* axis represents the −log_10_ *p*-value. Dotted lines indicate fold-change cutoffs (|log_2_(1.5)|) and *p*-value cutoff (−log_10_(0.05)). Black points on the left indicate down-regulated proteins; black points on the right indicate up-regulated proteins; gray points indicate proteins without significant differential expression. Selected top up-regulated (red boxes) and down-regulated (blue boxes) proteins are marked within the graph. (**D**) Representative Western blot depicting the expression of CD99, GPI, and IGFALS in EVs extracted from CD99pos and CD99neg TC-71 cells. GAPDH was used as loading control.

We previously demonstrated that EVs from CD99pos vs. CD99neg cells deliver a differential microRNAs cargo able to influence the phenotype of EWS recipient cells [20]. Here, we evaluated the consequences of CD99 modulation in the proteomics cargo of EVs. We compared the proteomic profile of CD99neg EVs versus CD99pos EVs (Figure 4A). On the basis of a *p*-value < 0.05, we identified a signature of 55 differentially expressed proteins (DEPs) (22 up-regulated and 33 down-regulated in CD99neg EVs; Figure 4B,C and Table 2).

Among the most up-regulated proteins in CD99neg EVs, we found complement C1q tumor necrosis factor-related protein 3 (C1QTNF3), retinol-binding protein 4 (RBP4), lactotransferrin (LTF), inter-alpha-trypsin inhibitor heavy chain H3 (ITIH3), insulin-like growth factor-binding protein complex acid labile subunit (IGFALS), antithrombin-II (SERPINC1), and inter-alpha-trypsin inhibitor heavy chain H1 (ITIH1). Among the most down-regulated proteins in CD99neg EVs, we found various histones, including HIST1H4A, HIST3H2A, HIST1H2BL, fibrinogen gamma chain (FGG), alpha and beta subunits of the protasome 20S the core complex (PSMA3, PSMB1, PSMA7, PSMA6, PSMA5, PSMA2, PSMB3, PSMA1, PSMB5, PSMA4), TNC, and TNXB. Western blotting analysis showed the down-regulation of CD99, as previously demonstrated [18], and confirmed the up-regulation of GPI and IGFALS in CD99neg compared to CD99pos EVs (Figure 4D).

**Figure 5:**
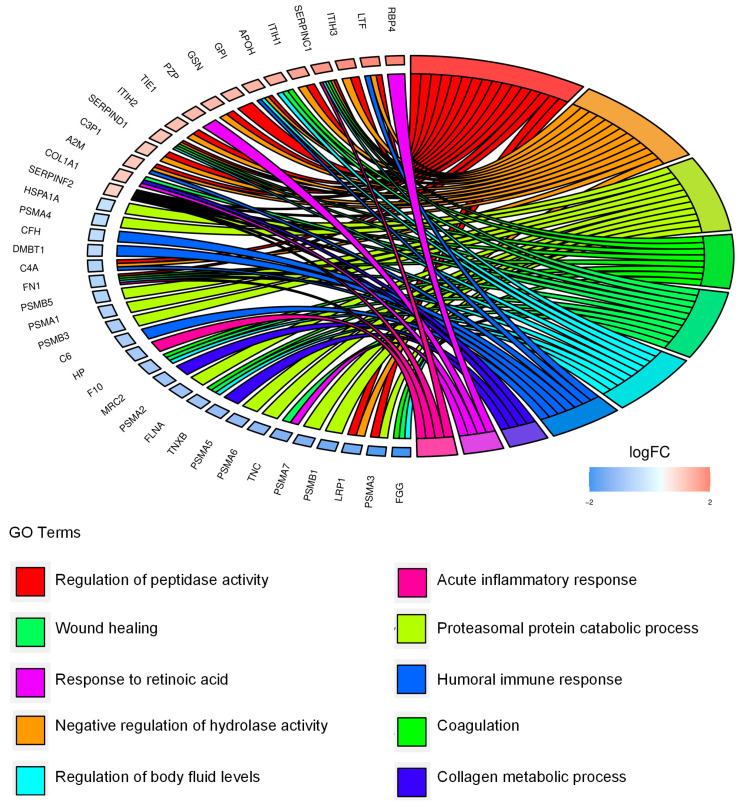
Extracellular CD99-associated protein signature points to migration, immunomodulation, and the extracellular proteasome. Chord diagram displays the relationship between the 55 differentially expressed proteins in EVs from CD99pos versus CD99neg TC-71 cells and the most significant GO biological processes.

To functionally annotate our data, we performed functional enrichment analysis of the 55 EV-associated differentially expressed proteins and we identified the proteasomal catabolic process, wound healing, and humoral immune response among the most significant biological processes (Figure 5, Table S4).

**Figure 6:**
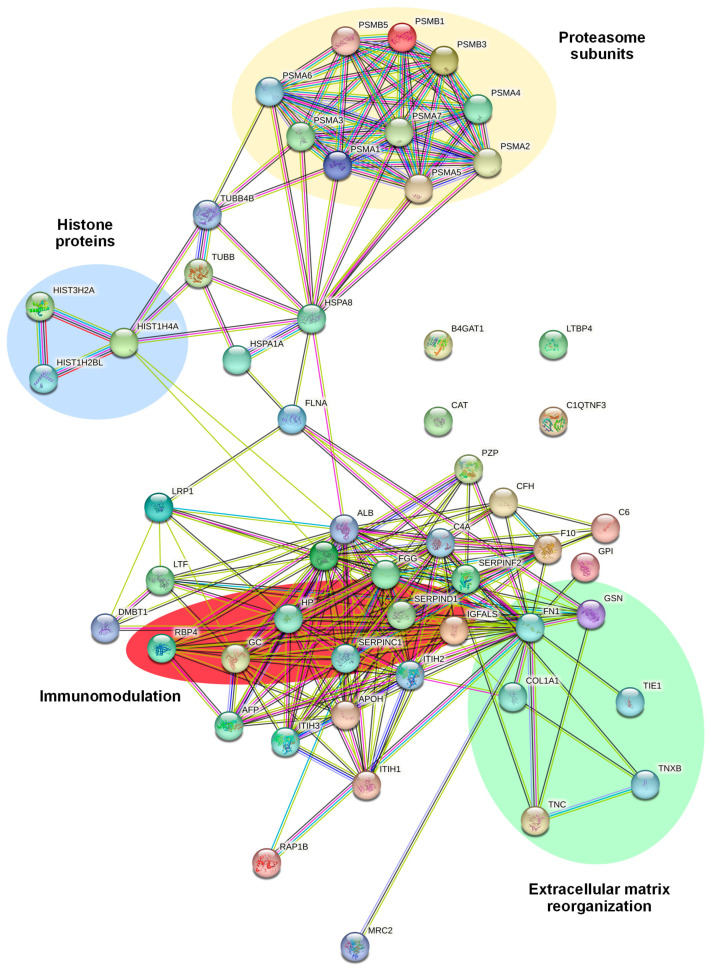
STRING analysis showing the predicted protein–protein interactions between the 55 differentially expressed proteins in EVs extracted from CD99neg versus CD99pos TC-71 cells. The protein nomenclatures and major networks of interactors are depicted.

We subsequently explored the potential interactions among the 55 DEPs. A network was developed using the STRING database (Figure 6). We observed four major networks of interactors: one displaying a hub of proteasome subunits, namely PSMA3, PSMB1, PSMA7, A6, A5, A2, B3, A1, B5, and A4; one cluster displaying a core of histone proteins, namely HIST1H4A, HIST3H2A, and HIST1H2BL; one cluster involved in extracellular matrix reorganization, including FN1, TNXB, and TNC; and one cluster of proteins involved in immunomodulation, including RBP4, SERPIND1, SERPINC1, and GC.

Overall, the results indicate that CD99neg and CD99pos EVs display differential proteomic profiles which are associated with migration, immunomodulation, and extracellular proteasomes.

## 3. Discussion

Intratumor heterogeneity refers to the presence of different tumor cell populations with varied molecular and phenotypic features in the same tumor specimen. Identifying the functional effectors of such tumor heterogeneity is challenging, but it promises to address unmet needs in cancer including the identification of biomarkers and advancements in diagnosis, risk-based stratification, and therapy. EWS is genetically homogeneous, but it is characterized by elevated epigenetic heterogeneity [26,27]. EWS::FLI1 is a major source of this heterogeneity, playing a significant role in the modulation of cellular transcriptome and proteome [4,26]. EWS cells dynamically fluctuate between a proliferative-prone EWS::FLI1 “high” state and a migration-prone EWS::FLI1 “low” state, according to the EWS::FLI1-mediated differential expression of proteins involved in actin cytoskeleton and cell–cell/cell–matrix interactions [4]. However, other factors collaborate with EWS::FLI1 to regulate phenotypic landscape of EWS as well as clinical presentation, therapeutic response, and patient outcomes [6,28,29,30,31]. In this study, we focused on CD99, a membrane-bound glycoprotein controlled by EWS::FLI1, routinely used to diagnose EWS [10]. Taking advantage of a CD99 knockdown cellular model and differential mass spectrometry analysis, we demonstrated that CD99-negative cells and related EVs display compartment-specific proteomic signatures which still converge to the up- or down-regulation of proteins relevant to cell migration and immunomodulation.

Proteomics represents a valuable strategy to close the gap between the genome and phenotype. Comparative multi-omics analyses between transcriptomic and proteomic data demonstrate a low global correlation between the levels of mRNA and proteins in tumor cells [32,33]. Accordingly, proteomics is gaining attention to understand the complexity of cancer biology and for the identification of novel markers of diagnosis, prognosis and therapeutic targets not yet uncovered by genomic or transcriptomic approaches [34]. This could be especially significant for cancer subtypes characterized by genetic stability, including EWS. Two-dimensional difference gel electrophoresis (2D DIGE) and MS performed in a cohort of EWS biopsies identified the protein nucleophosmin as differentially expressed between good- and poor-prognosis samples [35]. A recent surfaceome and global proteome analysis in EWS patients-derived cell lines identified known EWS-associated proteins including IL1RAP, CD99, STEAP1, and ADGRG2, as well as new potential cell surface targets, including ENPP1 [36]. Another proteomic profiling in EWS cell lines and clinical specimens of EWS cells and their EVs identified the UGT3A2 protein as being highly specific to EWS cells and their EVs compared to control samples [37]. For cancer biology, our functional annotation analyses demonstrate that CD99-negative cells and related EVs are characterized by a migration-suppressive, pro-immunostimulatory proteomic profile compared to their CD99-positive counterparts. Among the up-regulated proteins in cells were TMSB4X, which sequesters actin monomers, thus inhibiting actin polymerization [38], EZR, a cytoplasmic peripheral membrane protein which serves as a linker between the plasma membrane and the actin cytoskeleton [39], XAGE1, a testis cancer antigen characterized by a strong immunogenicity able to induce CD4 and CD8 T lymphocyte responses in cancer [40], and the HLA class I histocompatibility antigen, A alpha chain, which is involved in the presentation of intracellular antigens by tumor-specific cytotoxic CD8 T lymphocytes [41]. Among the up-regulated proteins in EVs were ITIH1, 2, and 3 proteins, belonging to the family of inter-α-trypsin protease inhibitors, which stabilize the extracellular matrices by binding hyaluronic acid, thus exerting anti-metastatic effects in cancer [42], and RBP, which stimulates macrophages’ polarization in cancer models [43]. Data from the literature support the utility of these proteins in driving clinical decision making for patient care in EWS, supporting their use as possible EWS biomarkers. High ezrin intensity established by immunohistochemistry significantly correlated with better 5-year event-free survival in a cohort of 53 newly diagnosed EWS patients [39]. High levels of XAGE1 have been detected in the majority of EWS cases [44,45], and XAGE1 has been considered for the development of gene-modified receptor T cells against EWS [46]. Positive expression of HLA class I evaluated through immunohistochemistry correlates with a good prognosis in EWS patients [46].

For circulating biomarkers, EV cargo represents a bulk of molecules that may be exploited as non-invasive approaches to monitor disease progression in cancer [47]. We have previously demonstrated that EVs derived from CD99-positive or CD99-negative cells hold a differential microRNAs cargo that influences the behavior of tumor recipient cells [20]. In addition, we demonstrated that the delivery of CD99-positive EVs maintains the undifferentiated, pro-migrative status of EWS cells [20]. In this study, the characterization of the proteomic cargo of CD99-positive EVs led to the identification of potential new biomarkers of malignancy, such as (i) alpha and beta subunits of the proteasome 20S core complex (PSMA3, PSMB1, PSMA7, PSMA6, PSMA5, PSMA2, PSMB3, PSMA1, PSMB5, PSMA4), which catalyze the degradation of obsolete or damaged endogenous proteins downstream of the ubiquitination process [48,49,50]; (ii) histones, including HIST1H4A, HIST3H2A, HIST1H2BL, that serve as the structural scaffold for the organization of nuclear DNA into chromatin [51]; and (iii) TNC and TNXB, two members of the tenascin family of extracellular matrix glycoproteins displaying multifunctional pro-tumorigenic activities as major components of the extracellular matrix surrounding cancer cells [52]. Clinical application of the proteomic content of EWS EVs is only starting to be revealed [47]. The presence of CD99 in EVs from EWS cells has been already established [18,20,53]. Data from the literature support the clinical utility for some of the EV-associated proteins identified in this study in EWS. TNC positivity assessed via immunohistochemistry correlates with a poor survival rate in EWS patients [54]. On the other side, preclinical evidence supports the efficacy of the 20S proteasome inhibitors in in vitro and in vivo EWS models, suggesting that the proteasome 20S core complex might represent a biomarker of response to these agents [55].

As a major limitation of this study, the evidence that we provide is based on a single cellular model. Therefore, the use of additional cellular models and analysis in clinical specimens, including tissues and plasma samples, would be beneficial to address the exact biological and clinical value of our findings. For clinical samples, the use of antibody-based approaches including immunohistochemistry and the ELISA assay, for cellular and circulating biomarkers [56,57], respectively, represents a concrete opportunity to measure protein expression levels and execute correlation analyses with patients’ survival and clinical–pathological parameters.

## 4. Materials and Methods

### 4.1. Cell Lines

TC-71 cells were kindly provided by T.J. Triche (Children’s Hospital, Los Angeles, CA, USA). Stable CD99-silenced cells were generated from the TC-71 cell line transfected with shRNA targeting the 3′ untranslated region of CD99 (called CD99neg) or a scrambled shRNA control (called CD99pos), as previously described [17,18]. The cells were cultured in Iscove’s modified Dulbecco’s medium (IMDM; EuroClone, Milan, Italy) enriched with 10% fetal bovine serum (FBS) (EuroClone) supplemented with 100 U/mL penicillin and 100 mg/mL streptomycin and incubated at 37 °C in a humidified atmosphere at 5% CO_2_. CD99pos/neg cellular variants were maintained in a regular culture medium supplemented with neomycin (500 μg/mL, G5013, Sigma-Aldrich, St. Louis, MO, USA). Cell lines were tested for mycoplasma contamination (MycoAlert Mycoplasma Detection Kit, Lonza, Walkersville, MD, USA) and authenticated by means of short tandem repeat (STR) polymerase chain reaction (PCR) analysis (CLA service by Eurofins Genomics; last control July 2023).

### 4.2. Isolation and Characterization of EVs

EVs were isolated from cell culture medium with ExoQuick-TC (EQ, System Biosciences) according to the manufacturer’s instructions. Briefly, cells were cultured for 24 h in IMDM supplemented with EV-depleted FBS. The serum was depleted of bovine EVs by ultracentrifugation at 100,000× *g* for 6 h, followed by filtration through a 0.2 µm filter prior to use. The conditioned medium was centrifuged at 3000× *g* for 15 min to remove cell debris. The supernatant was transferred to a new sterile tube and EQ precipitation solution was added for an overnight incubation at +4 °C. EVs were isolated the day after by means of centrifugation at 1500× *g* for 30 min at room temperature (RT). EV pellets were resuspended in phosphate-buffered saline (PBS). PD Spin Trap G-25 columns (Cytiva, Ge-Healthcare, Marlborough, MA, USA) were used for desalting, buffer exchange and sample clean-up. Small molecules like salt, free labels or other impurities were efficiently separated from the high-molecular-weight substances of interest for proteomics analysis. The protein concentration of EVs was determined using a protein assay kit (Bio-Rad). Characterization of EVs (concentration, size and markers) was performed as previously described [20]. Briefly, we employed the NanoSight NS300 system (NanoSight technology, Malvern, UK) to evaluate the concentration and size of isolated EVs. We employed Western blot to evaluate the EV markers TSG101 and ALIX. The following antibodies were used: anti-TSG101 (4A10) (GTX70255; GeneTex) and anti-Alix 3A9 (MA1-83977; Thermo Fisher Scientific, Waltham, MA, USA).

### 4.3. Western Blotting

Lysis of cells and EVs was performed using the radioimmunoprecipitation assay (RIPA) buffer (89900, Thermo Fisher Scientific) supplemented with protease and phosphatase inhibitors (A32959, Thermo Fisher Scientific). An equal amount of proteins were run on SDS gels under denaturing conditions and blotted onto nitrocellulose membranes. The membranes were incubated overnight at 4 °C with the following primary antibodies: anti-CD99 (sc-53148; Santa Cruz Biotechnology, Dallas, TX, USA), anti-Ezrin (E8897; Sigma-Aldrich); anti-XAGE1A (A61802; Epigentek, Farmingdale, NY); anti-GAPDH (5174; Cell Signaling); anti-GPI (MA515396; Thermo Fisher Scientific), anti-IGFALS (sc-377131; Santa Cruz Biotechnology).

### 4.4. Mass Spectrometry (MS)

#### 4.4.1. Sample Preparation

Cells were collected, washed, lysed with RIPA buffer and sonicated. EVs were lysed with RIPA buffer and sonicated too. Proteins were then precipitated with cold acetone and resuspended. Proteins were then reduced in 25 µL of 100 mM NH4HCO3 with 2.5 μL of 200 mM DTT (Merck, Rahway, NJ, USA) at 60 °C for 45 min and next alkylated with 10 μL of 200 mM iodoacetamide (Merck) for 1 h at RT in dark conditions. Iodoacetamide excess was removed by the addition of 200 mM DTT. Proteins were then digested with 2 μg of Trypsin (Sigma-Aldrich) overnight at 37 °C. The digests were dried using a Speed Vacuum and then desalted [58].

#### 4.4.2. Proteomic Analysis

Digested peptides were analyzed with an UHPLC Vanquish system (Thermo Fisher Scientific) coupled with an Orbitrap Q-Exactive Plus (Thermo Fisher Scientific). Peptides were separated by a reverse phase column (Accucore™ RP-MS 100 mm × 2.1 mm, particle size 2.6 µm). The column was maintained at a constant temperature of 40 °C at a flow rate of 0.200 mL/min. Mobile phase A and B were water and acetonitrile, respectively, both acidified with 0.1% formic acid. The analysis was performed using the following gradient: 0–5 min from 2% to 5% B; 5–55 min from 5% to 30% B; 55–61 from 30% to 90% B and held for 1 min; and at 62.1 min, the percentage of B was set to the initial condition of the run at 2% and held for about 8 min in order to re-equilibrate the column, for a total run time of 70 min. The mass spectrometry analysis was performed in positive ion mode. The ESI source was used with a voltage of 2.8 kV. The capillary temperature, sheath gas flow, auxiliary gas and spare gas flow were set at 325 °C, 45 arb, 10 arb and 2, respectively. The S-lens was set at 70 rf. For the acquisition of spectra, a data-dependent (ddMS2) top 10 scan mode was used. Survey full-scan MS spectra (mass range *m*/*z* 381 to 1581) were acquired with resolution R = 70,000 and AGC target 3 × 10^6^. Tandem MS (MS/MS) fragmentation was performed using high-energy c-trap dissociation (HCD) with resolution R = 35,000 and AGC target 1 × 10^6^. The normalized collision energy (NCE) was set to 30. The injection volume was 3 μL. The mass spectra analysis was carried out using MaxQuant software (version 1.6.14). MaxQuant parameters were set as follows: trypsin was selected for enzyme specificity; the search parameters were fixed to an initial precursor ion tolerance of 10 ppm and MS/MS tolerance of 20 ppm; carbamidomethylation was set as a fixed modification, whereas oxidation was set as a variable modification. The maximum missed cleavage was set to 2. The Andromeda search engine was used to search the spectra in MaxQuant against the Uniprot_CP_Human_2018 sequence database. Label-free quantification was performed, including a match between the run option and the following parameters: the protein and peptide false discovery rate was set to 0.01; the quantification was based on the extracted ion chromatograms, with a minimum ratio count of 1; and the minimum required peptide length was set to 7 amino acids. Data are available via ProteomeXchange with the identifier PXD046846.

### 4.5. Label-Free Proteomics Quantification and Differential Expression (Analysis of MS Data)

Data were analyzed using the R package DEP (1.16.0), integrated within the Bioconductor project version 3.14 [59]. We retained those proteins that were identified (with LFQ intensity > 0) in all replicates of at least one condition. Data were transformed and normalized by means of variance stabilizing normalization [60]. To perform statistical analysis, data were imputed for missing values using random draws from a Gaussian distribution centered around a minimal value (q = 0.01). Differential expression analysis was performed by applying empirical Bayes moderated t-statistics on protein-wise linear models using limma [61]. Differentially expressed proteins were those with FDR ≤ 0.05 or *p*-values ≤ 0.05 for cells or EVs comparisons, respectively, and |log_2_(Fc)| ≥ log_2_(1.5). Gene Ontology and pathway analyses were performed using the Bioconductor software package clusterProfiler (4.2.2) in R [62]. For pathway analysis in clusterProfiler, significance for GO-BP terms was set to FDR-adjusted *p*-value < 0.05. For EV cargo analysis, within clusterProfiler, the simplify function (cutoff = 0.5, by = “Count”) was used to combine redundant terms from GO term enrichment results. Protein expression data were used for heatmap construction depicting normalized values scaled to the z-score with supervised hierarchical clustering for dendrogram construction applying calculated Euclidean distances. Data visualization was performed using R package ggplot2 (3.4.2) [63] and GOplot (1.0.2) [64]. Venn diagrams were constructed using the jvenn tool [65]. In order to predict protein–protein interactions, proteins were analyzed using STRING software version 12.0 (http://string-db.org) [66]. STRING analysis was performed by setting the species under investigation (*Homo sapiens*) with a medium confidence level (score 0.4) and medium FDR stringency.

## 5. Conclusions

In conclusion, fluctuations in CD99 levels in EWS cells and EVs are associated with compartment-specific proteomic expression profiles. The functional annotation of modulated proteins in cells and EVs points to cell migration, which is in line with the functional effects elicited by CD99 in EWS, and immunomodulation, which represents a new area of research to explore. The application of proteomic studies is at a relatively early stage, but it holds promises to improve our understanding of EWS heterogeneity and to favor the identification of novel biomarkers. Here, we offer a set of cellular and EV-associated circulating proteins derived from CD99-positive or CD99-negative EWS cells, which should be considered for further validation.

## Figures and Tables

**Table 1:** Differentially expressed proteins in CD99neg versus CD99pos TC-71 cells. The proteins were sorted according to fold change (log_2_).

**Up-Regulated Proteins in CD99neg Cells**			
**Accession Name**	**Name**	**Fold Change (log_2_)**	**FDR**
ALDH1A1	Aldehyde dehydrogenase 1A1	5.63	5.60 × 10^−14^
TMSB4X	Thymosin beta-4	4.97	4.63 × 10^−3^
NUP50	Nuclear pore complex protein Nup50	3.28	6.42 × 10^−3^
XAGE1	X antigen family member 1	3.2	5.60 × 10^−14^
RCN3	Reticulocalbin-3	2.26	5.60 × 10^−14^
HLA-A	HLA class I histocompatibility antigen, A alpha chain	1.88	1.43 × 10^−4^
STOM	Stomatin	1.44	6.38 × 10^−5^
LGALS1	Galectin-1 (Gal-1)	1.4	1.27 × 10^−11^
FTL	Ferritin light chain	1.35	3.40 × 10^−3^
SPR	Sepiapterin reductase	1.27	2.42 × 10^−2^
ARHGDIB	Rho GDP-dissociation inhibitor 2	1.18	1.17 × 10^−9^
NCAPG	Condensin complex subunit 3	1.15	1.32 × 10^−2^
HSPA1B	Heat shock 70 kDa protein 1B	1.11	2.46 × 10^−11^
CPNE3	Copine-3	0.941	3.34 × 10^−2^
RCN2	Reticulocalbin-2	0.941	5.01 × 10^−3^
HSPB1	Heat shock protein beta-1	0.914	2.92 × 10^−3^
CAPG	Macrophage-capping protein	0.832	2.54 × 10^−4^
ANXA1	Annexin A1	0.774	4.49 × 10^−4^
FH	Fumarate hydratase	0.768	1.04 × 10^−3^
SEPTIN2	Septin-2	0.758	1.12 × 10^−3^
EZR	Ezrin	0.712	1.12 × 10^−4^
NARS1	Asparagine–tRNA ligase, cytoplasmic	0.71	3.25 × 10^−7^
SYPL1	Synaptophysin-like protein 1	0.631	4.17 × 10^−5^
**Down-Regulated Proteins in CD99neg Cells**			
**Accession Name**	**Name**	**Fold Change (log_2_)**	**FDR**
CD99	CD99 antigen	−1.87	4.51 × 10^−2^
SPART	Spartin	−1.58	5.60 × 10^−14^
TPP2	Tripeptidyl-peptidase 2	−1.46	2.94 × 10^−6^
PLEC	Plectin	−1.45	1.10 × 10^−2^
UCHL1	Ubiquitin carboxyl-terminal hydrolase isozyme L1	−1.16	2.31 × 10^−6^
PRPH	Peripherin	−1.12	4.35 × 10^−4^
PFN2	Profilin-2	−1.1	1.71 × 10^−2^
SUGT1	Protein SGT1 homolog	−1.08	5.60 × 10^−14^
UFM1	Ubiquitin-fold modifier 1	−1.08	8.38 × 10^−4^
CRIP2	Cysteine-rich protein 2	−1.01	7.55 × 10^−3^
ATAD3A	ATPase family AAA domain-containing protein 3A	−0.984	1.20 × 10^−2^
ARL3	ADP-ribosylation factor-like protein 3	−0.913	1.63 × 10^−2^
TUBB3	Tubulin beta-3 chain	−0.892	5.47 × 10^−5^
MIF	Macrophage migration inhibitory factor	−0.863	7.41 × 10^−5^
RANBP1	Ran-specific GTPase-activating protein	−0.838	1.38 × 10^−5^
ESD	S-formylglutathione hydrolase	−0.823	3.13 × 10^−3^
ISYNA1	Inositol-3-phosphate synthase 1	−0.814	2.25 × 10^−2^
NLN	Neurolysin, mitochondrial	−0.808	7.46 × 10^−5^
ATXN10	Ataxin-10	−0.778	4.27 × 10^−4^
IPO5	Importin-5	−0.753	2.58 × 10^−2^
HMGB1	High mobility group protein B1	−0.717	3.95 × 10^−2^
FSCN1	Fascin	−0.697	1.08 × 10^−3^
KPNA3	Importin subunit alpha-4	−0.685	1.57 × 10^−2^
ANXA7	Annexin A7	−0.679	1.55 × 10^−3^
SF3B6	Splicing factor 3B subunit 6	−0.668	1.42 × 10^−2^
G3BP2	Ras GTPase-activating protein-binding protein 2	−0.655	3.52 × 10^−2^
PITPNB	Phosphatidylinositol transfer protein beta isoform	−0.654	8.42 × 10^−3^
TPT1	Translationally controlled tumor protein	−0.625	3.06 × 10^−2^
PYCR1	Pyrroline-5-carboxylate reductase 1, mitochondrial	−0.608	4.07 × 10^−7^
EIF4E	Eukaryotic translation initiation factor 4E	−0.603	2.84 × 10^−2^
ABCE1	ATP-binding cassette sub-family E member 1	−0.587	1.71 × 10^−3^

**Table 2:** Differentially expressed proteins in EVs from CD99neg versus CD99pos TC-71 cells. The proteins were sorted according to fold change (log_2_).

**Up-Regulated Proteins in CD99neg EVs**			
**Accession Name**	**Name**	**Fold Change (log_2_)**	***p*-Value**
C1QTNF3	Complement C1q tumor necrosis factor-related protein 3	1.66	6.94 × 10^−3^
RBP4	Retinol-binding protein 4	1.5	8.04 × 10^−3^
LTF	Lactotransferrin	1.43	2.00 × 10^−6^
ITIH3	Inter-alpha-trypsin inhibitor heavy chain H3	1.38	2.22 × 10^−8^
IGFALS	Insulin-like growth factor-binding protein complex acid labile subunit	1.34	3.44 × 10^−4^
SERPINC1	Antithrombin-II	1.31	1.19 × 10^−5^
ITIH1	Inter-alpha-trypsin inhibitor heavy chain H1	1.17	1.52 × 10^−5^
AFP	Alpha-fetoprotein	1.09	1.86 × 10^−5^
APOH	Beta-2-glycoprotein 1	1.03	4.85 × 10^−2^
CAT	Catalase	1.02	3.89 × 10^−3^
GC	Vitamin D-binding protein	1.02	2.54 × 10^−4^
GPI	Glucose-6-phosphate isomerase	0.94	1.64 × 10^−6^
GSN	Gelsolin	0.921	1.56 × 10^−8^
ALB	Albumin	0.908	6.22 × 10^−5^
PZP	Pregnancy zone protein	0.892	6.90 × 10^−7^
TIE1	Tyrosine-protein kinase receptor Tie-1	0.878	1.24 × 10^−2^
ITIH2	Inter-alpha-trypsin inhibitor heavy chain H2	0.832	1.31 × 10^−5^
SERPIND1	Heparin cofactor 2	0.802	4.36 × 10^−8^
C3P1	Putative protein C3P1	0.713	8.13 × 10^−3^
A2M	Alpha-2-macroglobulin	0.71	1.33 × 10^−4^
COL1A1	Collagen alpha-1(I) chain	0.71	4.51 × 10^−4^
SERPINF2	Alpha-2-antiplasmin	0.596	1.30 × 10^−4^
**Down-Regulated Proteins in CD99neg EVs**			
**Accession name**	**Name**	**Fold Change (log_2_)**	***p*-Value**
HIST1H4A	Histone H4	^−2^.55	2.71 × 10^−8^
HIST3H2A	Histone H2A type 3	−2.49	3.65 × 10^−8^
FGG	Fibrinogen gamma chain	−2.16	2.07 × 10^−3^
PSMA3	Proteasome subunit alpha type-3	−2.02	3.56 × 10^−5^
LRP1	Pro-low-density lipoprotein receptor-related protein 1	−1.74	6.28 × 10^−3^
PSMB1	Proteasome subunit beta type-1	−1.72	3.42 × 10^−4^
PSMA7	Proteasome subunit alpha type-7	−1.61	1.19 × 10^−4^
TNC	Tenascin	−1.58	2.72 × 10^−4^
PSMA6	Proteasome subunit alpha type-6	−1.43	1.11 × 10^−3^
PSMA5	Proteasome subunit alpha type-5	−1.37	5.83 × 10^−5^
HIST1H2BL	Histone H2B type 1-L	−1.34	1.39 × 10^−5^
TNXB	Tenascin-X	−1.33	8.98 × 10^−8^
RAP1B	Ras-related protein Rap-1b	−1.27	2.65 × 10^−2^
FLNA	Filamin-A	−1.2	8.78 × 10^−4^
PSMA2	Proteasome subunit alpha type-2	−1.19	5.16 × 10^−3^
MRC2	C-type mannose receptor 2	−1.16	2.00 × 10^−2^
F10	Coagulation factor X	−1.11	4.64 × 10^−2^
HP	Haptoglobin	−1.07	9.35 × 10^−9^
TUBB4B	Tubulin beta-4B chain	−1.06	4.10 × 10^−5^
C6	Complement component C6	−1.04	7.49 × 10^−5^
LTBP4	Latent-transforming growth factor beta-binding protein 4	−1.04	7.37 × 10^−3^
PSMB3	Proteasome subunit beta type-3	−1.03	6.70 × 10^−3^
PSMA1	Proteasome subunit alpha type-1	^−0^.941	3.64 × 10^−3^
PSMB5	Proteasome subunit beta type-5	−0.904	1.85 × 10^−3^
FN1	Fibronectin	−0.9	5.22 × 10^−10^
C4A	Complement C4-A	−0.89	2.45 × 10^−5^
B4GAT1	Beta-1,4-glucuronyltransferase 1	−0.816	1.78 × 10^−2^
HSPA8	Heat shock cognate 71 kDa protein	−0.816	3.63 × 10^−4^
DMBT1	Deleted in malignant brain tumors 1 protein	−0.766	3.36 × 10^−2^
TUBB	Tubulin beta chain	−0.744	3.29 × 10^−3^
CFH	Complement factor H	−0.714	2.93 × 10^−5^
PSMA4	Proteasome subunit alpha type-4	−0.711	3.65 × 10^−3^
HSPA1A	Heat shock 70 kDa protein 1A	−0.633	3.32 × 10^−4^

## Data Availability

The original contributions presented in the study are included in the article/Supplementary Materials. Further inquiries can be directed to the corresponding authors. Proteomics data are available via ProteomeXchange with identifier PXD046846.

## Supplementary Materials

The supporting information can be downloaded at: https://www.mdpi.com/article/10.3390/ijms25031588/s1.
