# Supplementary material for: CD99 Modulates the Proteomic Landscape of Ewing Sarcoma Cells and Related Extracellular Vesicles
Source: Int J Mol Sci. 2024 Jan 27;25(3):1588. doi: 10.3390/ijms25031588 (PMC10855178; doi:10.3390/ijms25031588)
Supplement: Supplementary file 1 [file ijms-25-01588-s001.zip › Figure S1.pdf]

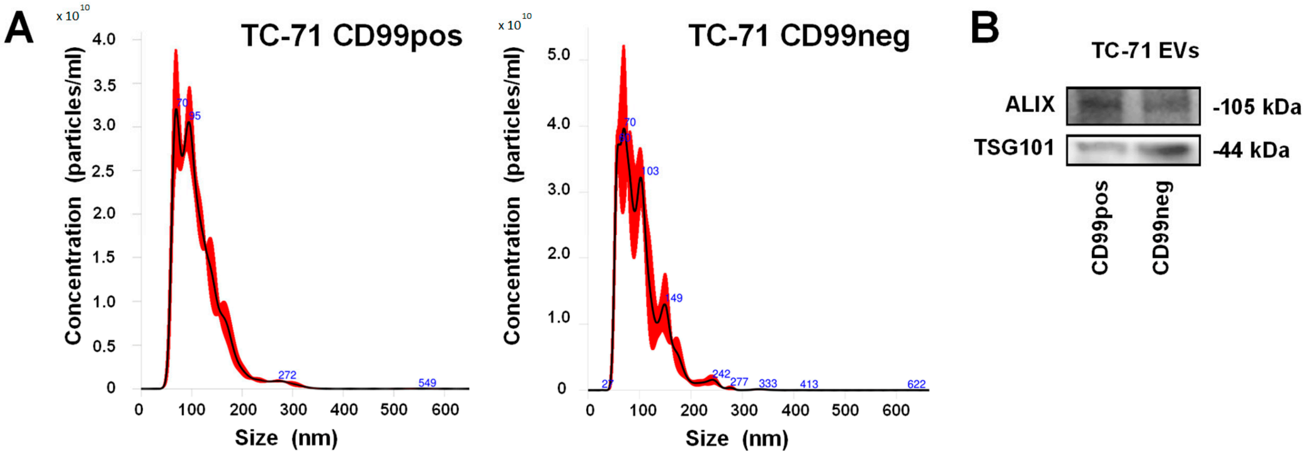

Supplementary Figure S1. (**A**) Nanoparticle tracking analysis of size distribution and concentration of EVs from TC-71 CD99pos (mean nm diameter (nm): 112.7 ± 2.9; concentration: $2.53 \times 10^{12} \pm 1.90 \times 10^{11}$) and TC-71 CD99neg (mean diameter (nm): 101.3 ± 0.6; concentration: $2.92 \times 10^{12} \pm 1.48 \times 10^{11}$) cells. (**B**) Western blot depicting the expression of EVs markers ALIX and TSG101 in EVs extracted from TC-71 CD99pos and TC-71 CD99neg cells.
